# In Situ Low-Temperature Carbonization Capping of LiFePO_4_ with Coke for Enhanced Lithium Battery Performance

**DOI:** 10.3390/molecules28166083

**Published:** 2023-08-16

**Authors:** Fei Guo, Xiaoqi Huang, Yudong Li, Shaohui Zhang, Xiong He, Jinghua Liu, Zhiqiang Yu, Feng Li, Baosheng Liu

**Affiliations:** 1 School of Electronic Engineering, Guangxi University of Science and Technology, Liuzhou 545006, Chinaliubaosheng@gxust.edu.cn (B.L.); 2 Key Laboratory of Bio-Based Material Science & Technology of Ministry of Education, Northeast Forestry University, Harbin 150040, China

**Keywords:** LiFePO_4_, carbon coating, lithium battery, ultra-long cycle life, Coke

## Abstract

Lithium batteries incorporating LiFePO_4_ (LFP) as the cathode material have gained significant attention in recent research. However, the limited electronic and ionic conductivity of LFP poses challenges to its cycling performance and overall efficiency. In this study, we address these issues by synthesizing a series of LiFePO_4_/carbon (LFP/C) composites through low-temperature carbonization coating of LFP in the presence of Coke as the carbon source. The resulting lithium batteries utilizing LFP/C as the cathode material exhibited impressive discharge specific capacities of 148.35 mA·h/g and 126.74 mA·h/g at 0.1 C and 1 C rates, respectively. Even after 200 cycles of charging and discharging, the capacities remained remarkably high, with values of 93.74% and 97.05% retention, showcasing excellent cycling stability. Notably, the LFP/C composite displayed exceptional rate capability, and capacity retention of 99.27% after cycling at different multiplication rates. These findings underscore the efficacy of in situ low-temperature carbonization capping of LFP with Coke in significantly improving both the cycling stability and rate capability of lithium batteries.

## 1. Introduction

In recent years, the increasing demand for traditional energy sources in modern society has led to a growing interest in the development of low-cost, environmentally friendly electrochemical energy storage devices, such as aqueous zinc-ion batteries [1], lithium-ion batteries [2], zinc–air batteries [3], and so on, in order to achieve sustainable development [4]. Electrochemical energy storage is an emerging green power supply technology, with the characteristics of high energy conversion efficiency, long life, safety and reliability, pollution free, and energy-efficient energy storage, and has been used extensively in the past few years. Today, many domestic and international academics apply the electrochemical energy storage system to electricity systems, for the reason that electrochemical energy storage has the potential to address the problem of reactive power loss in the power grid, it has been the subject of much attention. Among these technologies, lithium-ion batteries have emerged as highly promising candidates for energy storage systems and power supplies, owing to their high operating voltage, high specific energy, long lifespan, absence of memory effect, and minimal environmental pollution [5]. A typical lithium-ion battery comprises cathode and anode materials, a separator, and an electrolyte, with the cathode material representing a significant portion of the battery cost and playing a crucial role in the overall electrochemical performance and safety of the battery [6].

LFP has garnered significant attention in research due to its appealing characteristics such as safety, abundant raw material sources, high theoretical capacity, and excellent cycling performance [7,8,9,10,11]. The growing electric vehicle (EV) and hybrid electric vehicle (HEV) markets have created promising opportunities for LFP-based power batteries [12,13,14,15]. However, the utilization of single LFP as the cathode material in lithium-ion batteries hampers the multiplication rate and cycling performance of the battery due to its low electronic conductivity and limited lithium-ion diffusion ability [16,17,18]. To address these challenges and enable practical applications of LFP in power batteries, various approaches including carbon coating, conductive ion doping, and morphology optimization have been employed [19]. Carbon coating, in particular, has demonstrated effectiveness in improving the electrical conductivity of LFP by establishing a conductive network and enhancing the electrical contact between LFP particles. It facilitates the formation of a well-defined carbon layer on the LFP surface, limiting particle size variation, promoting uniformity, and establishing a robust conductive network that mitigates electrode polarization. Consequently, carbon coating enhances the multiplication performance, cycle life, and electrical conductivity of LFP-based batteries [20]. Due to its effectiveness and affordability, carbon coating has emerged as a promising and practical modification method for LFP cathode materials [21]. In Liu’s work [18], LiFePO_4_/C composites with several carbon contents were prepared by the carbothermal reduction method using glucose as the carbon source. The first discharge capacity of LFP/C (15% carbon content) was 160.7 mA·h/g at 0.1 C. The capacity retention rate after 100 cycles was still maintained at 82.1%. Raj [22] has fabricated carbon-coated LFP with citric acid as the carbon precursor by the sol–gel method. The sample with the stoichiometric ratio of metal ions to citric acid was 1:1, exhibited a discharge capacity of 148.2 mA·h/g (0.1 C) and 113.1 mA·h/g (5 C), while the capacity retention was as high as 96% after 300 cycles (1 C). The high rate capabilities and the capacity retention of LFP cells were significantly improved. It can be seen that the method of carbon coating has a positive effect on improving the performance of LFP materials.

The choice of suitable carbon sources in the carbon coating modification process significantly influences the enhancement of LFP electrochemical performance [8]. Different carbon sources exhibit distinct characteristics that impact the shape, structure, and coating of lithium iron phosphate particles during the carbonization process. Commonly used carbon sources in carbon capping include inorganic carbon sources (e.g., carbon black, carbon nanotubes, and graphene) [23,24,25], organic carbon sources (e.g., sucrose, glucose, and citric acid) [26,27,28], and organic polymer carbon sources formed through in situ polymerization of organic monomers (e.g., polyaniline, polyacrylic acid, and polyvinyl alcohol) [29,30]. In this study, Coke, a widely consumed beverage, was explored as a carbon source. Coke contains sugar, carbonated water (carbon dioxide and water), caramel, phosphoric acid, caffeine, and other components. Since its exact composition is still unknown and it has not been studied as a carbon source so far, this paper innovates by studying Coke as a carbon source. Furthermore, employing low-temperature synthesis during the in situ carbonization and encapsulation process can reduce the particle size of the obtained sample, as prolonged high-temperature calcination can lead to larger particle sizes [31]. The smaller particle size promotes the formation of a robust conductive network, diminishes electrode polarization effects, and improves the multiplication performance, cycle life, and conductivity of the LFP-based batteries.

In this study, LFP was first prepared by the conventional solid-phase method. And then, LFP/C composites were innovatively synthesized by in situ low-temperature carbon-coating with Coke as the carbon source. This carbon coating process was carried out under air and low-temperature (300 °C) conditions to form a uniform carbon layer on the surface of LFP/C composites with seldom Fe^2+^ oxidation, which was expected to improve the Coulombic efficiency and cycling stability. Afterward, the microcosmic appearance and microstructure were examined by SEM and XRD detections. The impact of in situ low-temperature carbon-coated by coke has been investigated by electrochemical tests including EIS, CV, and charge–discharge curves in detail.

## 2. Results and Discussion

### 2.1. Material Structure and Morphology Characterization Testing

**Figure 1:**
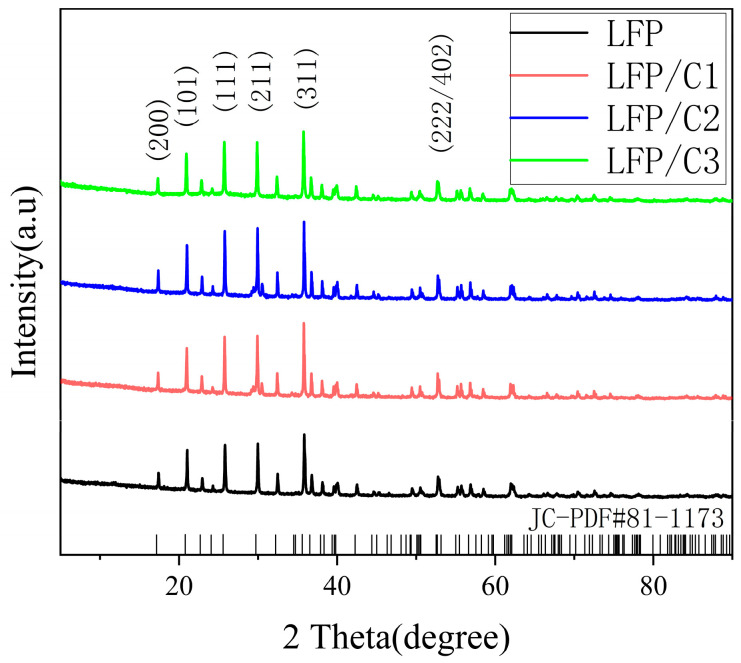
The XRD patterns of original LiFePO_4_ (LFP) and carbon-coated LiFePO_4_ (LFP/C1, LFP/C2, LFP/C3 means LFP with Coke added 10 mL, 20 mL, and 30 mL, respectively).

As shown in Figure 1, the X-ray diffraction (XRD) test results of four distinct cathode materials are compared with the standard LFP patterns. The XRD patterns of LFP/C1, LFP/C2, and LFP/C3, obtained through in situ low-temperature carbonization coating using Coke as the carbon source, exhibit similar peak shapes and positions to pure LFP, aligning with the *PDF#81-1173* standard card. No additional impurity peaks are observed, indicating that all four samples belong to the orthorhombic crystal structure. The high-intensity diffraction peaks, flat back bottoms, and narrow half-height widths in the XRD patterns suggest excellent crystallinity and high purity of the LFPs. The findings further reveal that the appropriate amount of Coke as the carbon source, used in the in situ low-temperature carbonization coating process, enhances the crystallinity and structural stability of the LFPs. Thus, the carbon coating process proves effective in improving the material properties of LFP, as confirmed by the XRD analysis.

**Figure 2:**
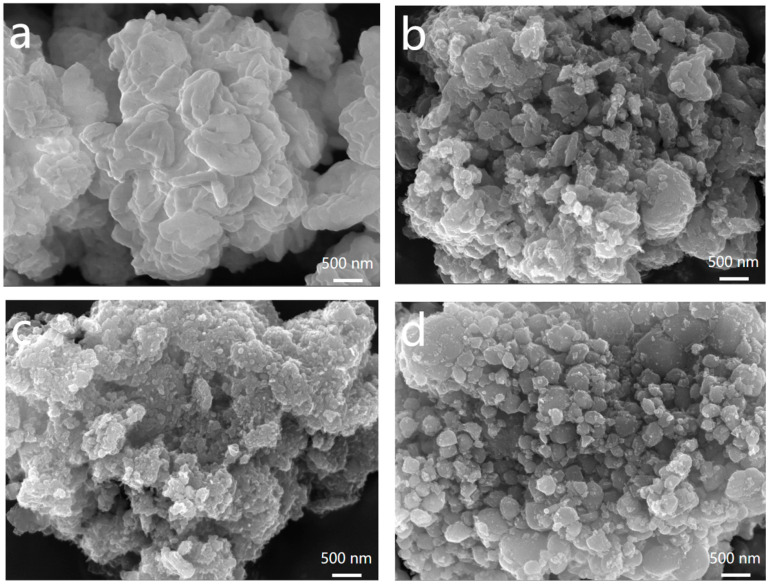
SEM images of materials: (**a**) pure LFP, (**b**) LFP/C1, (**c**) LFP/C2, and (**d**) LFP/C3.

The particle size of the cathode active material plays a crucial role in its electrochemical performance, with smaller particle sizes being favorable for lithium-ion diffusion. Conversely, larger particle sizes limit diffusion due to their smaller specific surface area, increased diffusion path growth, and elevated diffusion resistance [32,33]. Figure 2a–d displays the SEM images of LFP, LFP/C1, LFP/C2, and LFP/C3, respectively. The results indicate that the synthesized LFPs possess a nano-sized morphology both before and after carbon coating, with particle sizes ranging from 100 nm to 500 nm. Following carbon coating, the particle size reduces, accompanied by an increase in surface activity, resulting in enhanced repulsive forces between the particles. This leads to a more uniform particle distribution, with sequential decreases in particle size observed in LFP/C3, LFP/C1, and LFP/C2. The carbon coating process involves the deposition of a thin film of activated carbon on the newly crystallized LFP particles. This film restricts atomic diffusion during the calcination process and impedes particle growth, leading to an increased specific surface area [34,35]. Consequently, the electrochemical performance is influenced by the microstructural changes induced by in situ low-temperature carbonization using Coke as a carbon source. Moreover, the formation of a conductive network is promoted, contributing to enhanced performance.

### 2.2. Electrochemical Performance Testing

**Figure 3:**
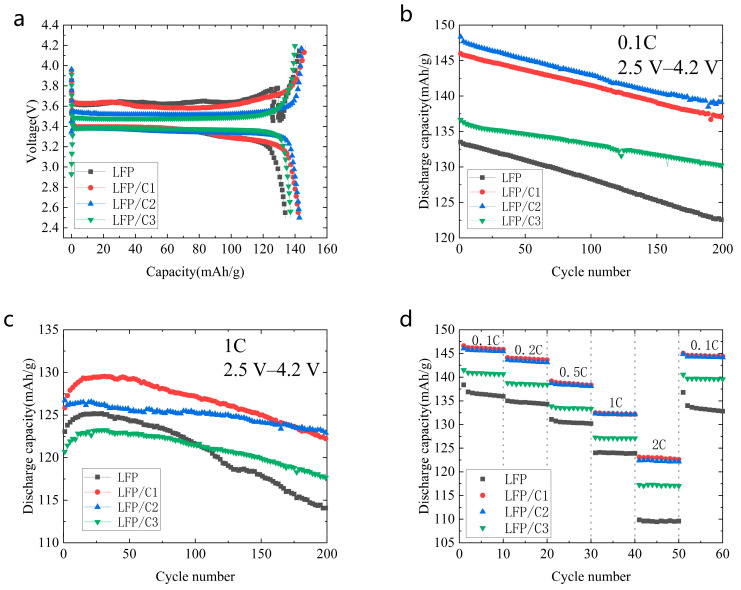
Charge–discharge curves of LFP along with LFP/C1, LFP/C2, and LFP/C3 cathode materials. (**a**) Initial charge/discharge curves; (**b**) 0.1 C cycling test; (**c**) 1 C cycling test; and (**d**) rate capability.

The electrochemical performance of four material groups, including pure LFP (LFP) and in situ low-temperature carbonization-coated LFPs (LFP/C1, LFP/C2, and LFP/C3) using Coke as the carbon source, was assessed through a charging and discharging cycle test in the voltage range of 2.5~4.2 V at 25 °C. Figure 3a displays the first charge–discharge curves, revealing notable improvements in both the first charge–discharge capacity and efficiency after carbon coating. The specific capacities of the first discharges were measured as 134.11 mA·h/g for LFP, 142.26 mA·h/g for LFP/C1, 142.70 mA·h/g for LFP/C2, and 137.37 mA·h/g for LFP/C3, accompanied by first charge/discharge efficiencies of 93.61%, 97.02%, 98.99%, and 98.16%, respectively. These results underscore the positive impact of in situ low-temperature carbonization capping with Coke on enhancing the electrical conductivity of the cathode materials. Remarkably, LFP/C2 exhibited the highest first charge–discharge capacity and efficiency among the investigated materials.

The cycling performance of lithium-ion batteries serves as a crucial indicator of their overall performance. Figure 3b,c depict the cycling performance of LFP, LFP/C1, LFP/C2, and LFP/C3 at multiplication rates of 0.1 C and 1 C, respectively. In Figure 3b, the first-cycle discharge specific capacities at a 0.1 C multiplication rate for LFP, LFP/C1, LFP/C2, and LFP/C3 are recorded as 133.52 mA·h/g, 145.99 mA·h/g, 148.35 mA·h/g, and 136.66 mA·h/g, respectively. After 200 cycles, the discharge capacities are measured as 122.50 mA·h/g, 137.16 mA·h/g, 139.08 mA·h/g, and 130.17 mA·h/g, with corresponding cycling capacity retention rates of 91.70%, 93.95%, 93.74%, and 95.12%, respectively. In the 1 C multiplication rate test, the first-cycle discharge-specific capacities for LFP, LFP/C1, LFP/C2, and LFP/C3 are 123.06 mA·h/g, 125.85 mA·h/g, 126.74 mA·h/g, and 120.70 mA·h/g, respectively. The discharge capacities after 200 cycles are 114.04 mA·h/g, 126.17 mA·h/g, and 114.04 mA·h/g, respectively. The cycling capacity retention rates for these materials are 92.67%, 97.09%, 97.05%, and 97.34%, respectively. Notably, LFP/C2 exhibits the highest discharge-specific capacity in both the 0.1 C and 1 C rate tests, indicating improved long-cycle stability of LFP cells after carbon coating at both low and high rates.

This enhancement in cycling performance can be attributed to several factors. The in situ carbonization capping process uniformly coats the LFP surface with a capping layer, while the transient high temperature generated during carbonization introduces oxygen vacancies into LFP. Ma et al. [36] induced the generation of surface oxygen vacancies by pre-embedding non-stoichiometric sodium ions instead of lithium on the surface of layered lithium-rich manganese-based cathode materials, which greatly facilitated the electrochemical activity of the transition metal elements and the diffusion rate of Li^+^, and ultimately achieved higher specific capacity, better rate capability, and smaller voltage degradation. Zhu et al. [37]. successfully introduced oxygen vacancies into LiMn_2_O_4_ cathode material by calcining it with a high-temperature shock process. The introduction of oxygen vacancies facilitates the diffusion of Li^+^ in the cathode material and improves the electrochemical performance of the cathode material. The carbon coating provides stable interfacial protection for the LFP, while the elevated temperature improves its electronic structure, enhancing carrier migration efficiency, reducing polarization effects, and improving Coulombic efficiency. The synergistic effect of these factors enables the ultra-long and stable cycling of the LFP. Coating LFP with an appropriate amount of carbon not only enhances its long-cycle stability but also improves specific discharge capacity.

Figure 3d illustrates the cycle test performance of the battery using different cathode materials, specifically LFP, LFP/C1, LFP/C2, and LFP/C3, under varying discharge rate conditions: 0.1 C, 0.2 C, 0.5 C, 1 C, and 2 C. The discharge-specific capacity exhibits a gradual decrease as the discharge rate increases. Higher discharge current density negatively impacts Li^+^ removal, leading to increased internal ohmic polarization and decreased battery performance. The discharge-specific capacity of LFP, LFP/C1, LFP/C2, and LFP/C3 at 0.1 C after 10 cycles is taken as the benchmark; when the discharge rate is gradually increased to 0.2 C, 0.5 C, 1 C, and 2 C, the capacity retention of LFP is 98.79%, 95.76%, 91.13%, and 80.61%, respectively. That of LFP/C1 is 98.50%, 94.86%, 90.64%, and 84.08%, respectively. Additionally, that of LFP/C2 is 98.41%, 94.99%, 90.89%, and 84.08%, respectively. Meanwhile, the capacity retention rate of LFP/C3 is 98.42%, 94.79%, 90.37%, and 83.21%, respectively. When the high-current discharge of 2 C was finished, and then recharged and discharged at a low current density of 0.1 C for 10 cycles, the capacity retention rates of LFP, LFP/C1, LFP/C2, and LFP/C3 were 97.69%, 99.00%, 99.15%, and 99.27%, respectively. It can be found that the capacity retention of LFP/C1, LFP/C2, and LFP/C3 after carbon coating is improved at a high rate, and the capacity retention of all is also better than that of pure LFP in the 0.1 C cycle after the high-rate cycling. This improvement can be attributed to in situ low-temperature carbonization and coating processes using Coke as the carbon source, which enhance the electronic structure of LFP, improve carrier migration efficiency, reduce polarization effects, and enhance rate capability.

**Figure 4:**
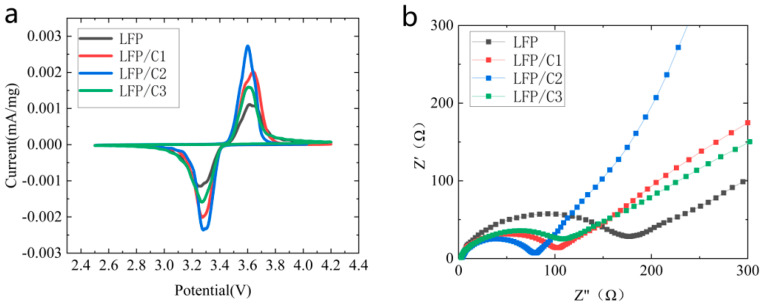
The results of the cyclic voltammetry (CV) test and AC impedance test conducted on LFP along with LFP/C1, LFP/C2, and LFP/C3 materials. (**a**) CV curve and (**b**) EIS curve.

In order to analyze the electrochemical performance of LFP, LFP/C1, LFP/C2, and LFP/C3 material samples more precisely, cyclic voltammetry tests (CV) were performed at a sweep rate of 0.1 mV/s and the test voltage range was set to 2.5–4.2 V. The results are presented in Figure 4a. This curve explores the effect of carbon coating on battery performance from a kinetic point of view. The oxidation and reduction peaks for all four sample groups are observed within the potential range of 3.1–3.8 V. The curves of the four materials exhibit similar patterns, featuring one oxidation peak and one reduction peak. The oxidation peaks and reduction peaks of LFP, LFP/C1, LFP/C2, and LFP/C3 occur at 3.634 V and 3.256 V, 3.615 V and 3.282 V, 3.600 V and 3.292 V, and 3.611 V and 3.270 V, respectively. The potential difference between the oxidation and reduction peaks of the first cycle, denoted as ΔE = |E_pa_ − E_pc_| (where E_pa_ and E_pc_ represent the oxidation and reduction peak potentials on the CV curve), is a measure of the potential difference [38]. The potential differences for LFP, LFP/C1, LFP/C2, and LFP/C3 are 0.378 V, 0.333 V, 0.308 V, and 0.341 V, respectively. Following carbon coating, the oxidation peak shifts to the left and the reduction peak shifts to the right, resulting in reduced ΔE. This indicates a decrease in electrode polarization, improved lithium-ion diffusion kinetics, enhanced reversibility, and reduced irreversible electrochemical reactions [22,39]. Notably, LFP/C2 exhibits the smallest potential difference, highest peak current, and largest integral area under the CV curve, indicating superior electrochemical capacity and optimal carbon content [18]. Thus, among the four material groups, LFP/C2 demonstrates the best electrochemical performance.

To further investigate the enhancement mechanism of the in situ low-temperature carbonization coating on the electrochemical performance of the LFP materials using Coke as a carbon source, subsequently, the activated LFP cells were subjected to AC impedance tests (EIS), after which the SEI film on the surface of each of the electrodes had been formed, and at this point, the AC impedance of the test was more reflective of the material’s ionic strength and charge transfer resistance. The EIS test results are shown in Figure 4b. The semicircle of the high intermediate frequency region in the figure represents the charge transfer impedance of the electrode, which is related to the insertion/removal process of lithium, and the smaller the radius, the smaller the impedance. The inclined line in the low-frequency region represents the diffusion resistance of Li^+^, and the larger the slope, the smaller the resistance [40,41,42]. As can be seen from the test results, the radius of the semicircle of LFP/C2 in the high-frequency region is smaller than that of LFP, LFP/C1, LFP/C3, and the slope of the slash line of LFP/C2 in the low-frequency region has the greatest slope, such that both the charge transfer impedance and the diffusional resistance of Li^+^ are minimized in LFP/C2.

The electrochemical properties of the materials determined by the study reported here were also compared to various previously reported LFP materials that were carbon-coated in a variety of different ways and with different carbon sources (as shown in Table 1). It can be clearly seen that the in situ low-temperature carbon-coated LFP with Coke as the carbon source has excellent global electrochemical properties, such as good discharge capacity and cycling stability, in comparison to previously reported materials.

As expected, after the mixing and drying of Coke with LiFePO_4_, the caramel-like substances and some additives were attached to the surface of LFP, which provided a physically isolated environment; secondly, the pyrolysis of the caramel-like substances provided a reducing environment, which could keep the divalent iron from oxidizing during the rapid low-temperature carbonization process, and, thus, the carbon-covered LFP could be prepared even when the immediate external environment was air, which could be proved by the decreasing impedance and increasing electro-activity of LFP/C.

## 3. Experiment

### 3.1. Materials Applied for the Experiment

FePO_4_ (99% purity, Shanghai Aladdin Biochemical Technology Co., Ltd., Shanghai, China), LiOH·H_2_O (analytical grade, Shanghai Aladdin Biochemical Technology Co., Ltd., Shanghai, China), Coke (The Coca-Cola Company., Ltd., Kunming, China), Super P conducting agent (battery grade, CyberElectrochemicals.com, accessed on 1 January 2023), PVDF (analytical grade, CyberElectrochemicals.com, accessed on 1 January 2023), N-Methylpyrrolidone (analytical grade, CyberElectrochemicals.com, accessed on 1 January 2023), LiPF_6_ lithium battery electrolyte (battery grade, Jiangxi Jinhui Lithium Materials Co., Ltd., Fuzhou, China), lithium flake (battery grade, Shenzhen Neware Electronics Co., Ltd., Shenzhen, China), Celgard 2400 diaphragm (battery grade, Celgard, Concord, NC, USA), gaskets, spring sheet (battery grade, Shenzhen Neware Electronics Co., Ltd.), CR2025 positive shell, negative shell (battery grade, Shenzhen Neware Electronics Co., Ltd.), and anhydrous ethanol (analytical grade, Chengdu Cologne Chemical Co., Ltd., Chengdu, China).

### 3.2. Synthesis of LFP/C

In the preparation of LFP powder, a solid-state method was employed. FePO_4_ and LiOH·H_2_O were mixed in a molar ratio of 1.05 (Li:Fe = 1.05:1). The mixture was then combined with an appropriate amount of anhydrous ethanol in a ball milling jar. The ball milling process involves rotating forward for 20 min and then reversing for 20 min at a speed of 400 rotations per minute (r/min), with 9 cycles. Following ball milling, the resulting mixture was dried at 60 °C, yielding the milled powder. Subsequently, the milled powder was subjected to sintering under an oxygen atmosphere using a tube furnace. Sintering was performed at 400 °C and 750 °C for 4 h and 8 h, respectively. After completion, the LFP powder was obtained upon cooling.

**Figure 5:**
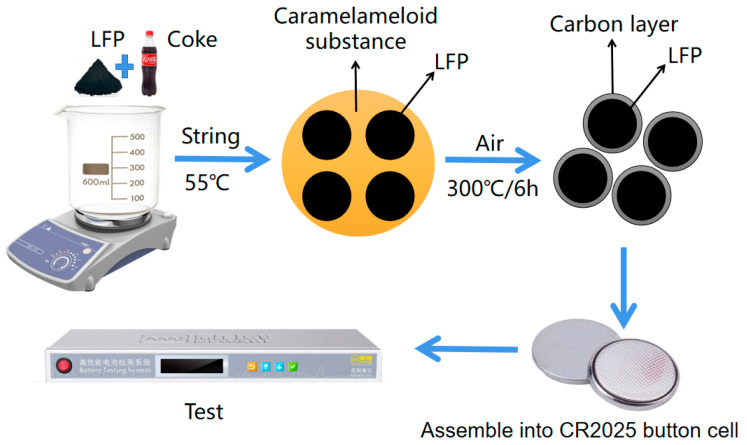
Flowchart of carbon-coated experiment, testing with Neware Battery Testing System BTS4000.

A predetermined quantity of LFP powder was thoroughly mixed with 10 mL, 20 mL, and 30 mL of Coke, respectively. Place the mixture on a magnetic stirrer and start heating and stirring, and then stop heating and stirring when the Coke is about to finish evaporating. The resulting mixture was then placed in a blast drying oven for 3 h to undergo a drying treatment, resulting in the formation of a dry powder mixture. Subsequently, the milled powder obtained earlier was transferred to a tube furnace and subjected to a heat treatment process. The powder was held at a temperature of 300 °C for 6 h under an air atmosphere. Following the heat treatment, the furnace was allowed to cool naturally. Finally, the material is thoroughly ground to 300 mesh using an agate mortar. This process led to the formation of LFP material, denoted as LFP/Cx (x = 1, 2, 3), which represents 10 mL, 20 mL, and 30 mL Coke addition during the LFP/C synthesis process, respectively. The experimental procedure is shown in Figure 5.

### 3.3. Preparation of Cathode Materials and Battery Assembly

Firstly, a mixture of PVDF (polyvinylidene fluoride) and N-methyl pyrrolidone was prepared in a ratio of 1:22. The mixture was heated and stirred to form a glue-like consistency. Subsequently, the cathode material, conductive agent Super P, and the prepared PVDF glue were combined in a ratio of 8:1:23. The mixture was thoroughly stirred to obtain a homogeneous slurry. This slurry was then uniformly coated onto a clean aluminum foil using a coating technique. Next, the coated foil was dried in a vacuum environment at 120 °C for 8 h to remove any remaining solvent and ensure proper adhesion. To enhance the electrode’s mechanical integrity, the dried foil was passed through a roller machine ten to twenty times, resulting in improved compaction and uniformity. Finally, the foil was cut into round sheets with a diameter of 12 mm using a sheet-cutting machine. These sheets were then dried again at 120 °C for 8 h under vacuum conditions to ensure complete removal of any remaining moisture. The resulting 12 mm diameter discs serve as the cathode electrodes, stored in a glove box with oxygen (O_2_) and water (H_2_O) content less than 0.1 parts per million (ppm), ready for further use in the battery assembly process.

The CR2025 button cell was assembled in a controlled environment within a glove box, where the O_2_ and H_2_O content were meticulously maintained below 0.1 ppm. The assembly process was carried out under an encapsulation pressure of 500 pounds per square inch (psi). The materials were carefully arranged in a specific order: starting with the negative case, followed by spring sheet, stainless steel tabs, lithium tabs, electrolyte, diaphragm, LFP positive electrode, and, finally, the positive case. To ensure proper insulation and prevent any potential short circuits, a double-layer diaphragm was utilized. The addition of the electrolyte was precisely controlled, with a volume of 50μL being added drop by drop. By adhering to these meticulous assembly steps and maintaining a controlled environment, the CR2025 button cell was successfully constructed and is now ready for further testing and evaluation.

### 3.4. Characterization of Materials

The crystal structure of the sample was determined through XRD (X-ray diffraction) analysis, providing insights into the qualitative and quantitative analysis of its physical phase components. The XRD measurements were performed using a Japan Rigaku SmartLab SE instrument, with a scanning range of 5° to 90° and a sweep speed of 5°/min. Cu-K α-rays were used as the light source, with a tube voltage of 40 kV and a tube current of 40 mA. The XRD scanning mode employed was continuous scanning. For the observation of surface morphology, smoothness, and particle size of the cathode material at the microscopic level, SEM (scanning electron microscope) analysis was conducted using a TESCAN MIRA LMS scanning electron microscope. This technique allowed for detailed examination and imaging of the sample’s surface characteristics, providing valuable insights into its microstructure.

### 3.5. Electrochemical Performance Measurements

The aged batteries underwent charge/discharge performance testing in a constant temperature box set at 25 °C. The Shenzhen Neware Battery Testing System BTS4000 was used as the testing instrument, with a voltage test range selected from 2.5 to 4.2 V. The cyclic charge/discharge test was conducted at a current of 0.1 C, where 1 C represents a rate of 150 mA·h/g. To assess the AC impedance (EIS) and cyclic voltammetry (CV), an electrochemical workstation (CHI760E) manufactured by Shanghai Chenhua Instrument Co. (Shanghai, China) was employed. The voltage range for these tests was set between 2.5 and 4.2 V. The AC impedance spectrum test utilized a frequency range of 0.01~100,000 Hz, an amplitude of 5 mV, and bias currents lower than 0.01 Hz. Fourier transform analysis was applied to extract meaningful data. The cyclic voltammetry test was performed with a scanning speed of 0.1 mV/s and five scanning segments were conducted.

## 4. Conclusions

In this study, pure LFP was synthesized using the high-temperature solid-phase method. Additionally, a range of LFP/C materials was prepared by performing in situ low-temperature carbonization coating of LFP in the presence of Coke as the carbon source. Scanning electron microscopy analysis revealed that an appropriate thickness of carbon coating effectively inhibited particle growth and improved the crystallinity and stability of the grain interface structure in LFP. In long-cycle tests at 0.1 C and 1 C discharge rates, LFP/C2 demonstrated specific capacities of 148.35 mA·h/g and 126.74 mA·h/g, respectively. After 200 cycles, the capacity retention rates were 93.74% and 97.05%, indicating that the suitable carbon coating significantly enhanced the stability and specific capacity of LFP during long cycling. Moreover, rate capability tests show that all carbon-coated LFP/C materials have improved capacity retention after different rate cycles and enhanced rate capability. Cyclic voltammetry and AC impedance tests further indicated that LFP/C2 possessed the smallest potential difference, lowest charge transfer impedance, and smallest Li^+^ diffusion resistance. In conclusion, lithium batteries employing LFP/C2 as the cathode material, with in situ low-temperature carbonization in the air using Coke as the carbon source, exhibited superior cycle stability and rate capability.

## Figures and Tables

**Table 1:** Comparison of the cathode performance of LFP with different coating materials and methods.

Carbon Source	Coating Method	Specific Capacity	Cycling Stability	Ref.
Glucose	Carbothermal reduction	160.7 mA·h/g (0.1 C)	82.1% (0.1 C, 100 cycles)	[18]
Citric acid	Sol–gel method	135.2 mA·h/g (1 C)	96% (1 C, 300 cycles)	[22]
Glucose	Co-precipitation	140.8 mA·h/g (0.1 C)	87.7% (0.1 C, 50 cycles)	[16]
Graphene nanosheet	Chemical vapor deposition	145 mA·h/g (0.1 C)	95.3% (0.1 C, 1000 cycles)	[43]
Graphene and sucrose	Solvothermal, drying, and calcination	163.7 mA·h/g (0.1 C)	97% (0.1 C, 30 cycles)	[44]
Sucrose	Hydrothermal method and heat treatment	128 mA·h/g (0.1 C)	No capacity fading (0.1 C, 50 cycles)	[45]
Coke	In situ low-temperature carbon coating	148.35 mA·h/g (0.1 C) 126.74 mA·h/g (1 C)	93.74% (0.1 C, 200 cycles) 97.05% (1 C, 200 cycles)	This work

## Data Availability

The data presented in this study are available on request from the corresponding author.

## Sample Availability

Samples of the compounds LFP and LFP/C are available from the authors.
